# Pharmacokinetics and Pharmacodynamics of the Nitroimidazole DNDI-0690 in Mouse Models of Cutaneous Leishmaniasis

**DOI:** 10.1128/AAC.00829-19

**Published:** 2019-08-23

**Authors:** Gert-Jan Wijnant, Simon L. Croft, Raul de la Flor, Mo Alavijeh, Vanessa Yardley, Stéphanie Braillard, Charles Mowbray, Katrien Van Bocxlaer

**Affiliations:** a London School of Hygiene & Tropical Medicine, Faculty of Infectious and Tropical Diseases, London, United Kingdom; b Pharmidex Pharmaceutical Services Ltd., London, United Kingdom; c Drugs for Neglected Disease initiative (DNDi), Geneva, Switzerland; d York Biomedical Research Institute, Department of Biology, University of York, York, United Kingdom

**Keywords:** cutaneous leishmaniasis, drug development, microdialysis, pharmacodynamics, pharmacokinetics, rate of kill, skin drug delivery

## Abstract

The nitroimidazole DNDI-0690 is a clinical drug candidate for visceral leishmaniasis (VL) that also shows potent *in vitro* and *in vivo* activity against cutaneous leishmaniasis (CL). To support further development of this compound into a patient-friendly oral or topical formulation for the treatment of CL, we investigated the free drug exposure at the dermal site of infection and subsequent elimination of the causative *Leishmania* pathogen. This study evaluates the pharmacokinetics (PK) and pharmacodynamics (PD) of DNDI-0690 in mouse models of CL. Skin microdialysis and Franz diffusion cell permeation studies revealed that DNDI-0690 permeated poorly (<1%) into the skin lesion upon topical drug application (0.063% [wt/vol], 30 μl). In contrast, a single oral dose of 50 mg/kg of body weight resulted in the rapid and nearly complete distribution of protein-unbound DNDI-0690 from the plasma into the infected dermis (ratio of the area under the curve [0 to 6 h] of the free DNDI-0690 concentration in skin tissue to blood [*f*AUC_0-6 h, skin tissue_/*f*AUC_0-6 h, blood_] is greater than 80%). Based on *in vivo* bioluminescence imaging, two doses of 50 mg/kg DNDI-0690 were sufficient to reduce the Leishmania mexicana parasite load by 100-fold, while 6 such doses were needed to achieve similar killing of L. major; this was confirmed by quantitative PCR. The combination of rapid accumulation and potent activity in the *Leishmania*-infected dermis indicates the potential of DNDI-0690 as a novel oral treatment for CL.

## Abstract (precis)

The nitroimidazole DNDI-0690 is a clinical drug candidate for visceral leishmaniasis (VL) that also shows potent *in vitro* and *in vivo* activity against cutaneous leishmaniasis (CL). To support further development of this compound into a patient-friendly oral or topical formulation for the treatment of CL, we investigated the free drug exposure at the dermal site of infection and subsequent elimination of the causative *Leishmania* pathogen.

## TEXT

Leishmaniasis is a poverty-associated infectious disease that has two main forms: visceral leishmaniasis (VL) and cutaneous leishmaniasis (CL). While VL is almost invariably fatal if left untreated, CL is not life-threatening but causes disfiguring skin lesions associated with severe social stigma and psychological morbidity (1). The different types of CL have a wide geographic distribution and vary in the causative *Leishmania* parasite species, which are transmitted to humans by infected female sand flies. In the Middle East, Old World Leishmania major and L. tropica CL commonly present as local papules, nodules, or ulcers that are mostly self-limiting but often leave lifelong scars on the exposed skin areas. After healing, L. tropica CL can relapse into a persisting, chronic form called leishmaniasis recidivans. In Central and South America, New World parasite species of the *Leishmania* subgenus, such as L. mexicana, generally cause mild forms of CL, while more complicated forms involving the mucous membranes of the nose, throat, and mouth are observed in patients infected with the *Viannia* subgenus, for example, L. braziliensis (2, 3). An additional cutaneous manifestation is post-kala azar dermal leishmaniasis (PKDL), a cutaneous sequela that can occur following the resolution of VL caused by L. donovani, characterized by widespread macular or papular lesions (3, 4). Worldwide, about 0.7 million to 1.2 million new cases of CL occur every year and about 1 billion people, mostly those living in resource-poor environments, are at risk (5). At present, treatment of CL is based on four drugs: pentavalent antimonials, miltefosine, amphotericin B, and paromomycin. All of these have well-documented limitations of effectiveness, toxicity, cost, or route of administration (6, 7). The Drugs for Neglected Diseases *initiative* (DND*i*), a nonprofit drug development partnership, has a strategy to deliver much-needed new drugs for CL. DND*i* defines the optimal target product profile (TPP) of a new chemical entity for CL as follows: (i) activity against all species of *Leishmania* causing CL (>15), (ii) a minimum 95% clinical efficacy and minimal scarring after accelerated healing of the skin lesions, (iii) use as an oral or topical formulation for a maximum of 7 or 14 days, respectively, (iv) well tolerated and safe in pregnancy, and (v) a cost under $5 per course (8). While topical formulations hold potential for the treatment of simple, self-healing lesions, oral drugs could be used for cases with a higher risk of parasite dissemination; both routes of administration avoid the need for painful drug injections, which are currently common (9).

**FIG 1:**
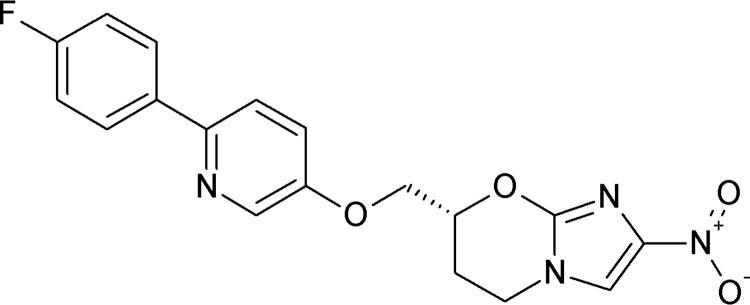
Chemical structure of DNDI-0690.

Nitroimidazoles are a medically important class of antimicrobial agents with a broad spectrum of activity, including against protozoan parasites, such as Trichomonas vaginalis, Trypanosoma cruzi, and *Giardia* (10). The prototype molecule for this class, metronidazole, was discovered in the 1950s, and in recent years, there has been a renewed interest in the therapeutic potential of nitroimidazoles, especially as novel antitubercular agents (11). Indeed, successful drug development efforts have resulted in the regulatory approval of delamanid (OPC-67683) for the treatment of multidrug-resistant tuberculosis (TB) by the European Medicines Agency (EMA) (12), while another bicyclic nitroimidazole compound, pretomanid (PA-824), is under investigation in phase III clinical trials (13). In 2010, DND*i* was granted access to a selected library of nitroimidazoles owned by the TB Alliance to speed up the development of novel therapies for neglected tropical diseases, including leishmaniasis. The antileishmanial activity of the nitroimidazooxazine DNDI-0690 (Fig. 1) was first discovered in 2015; it is a structural analogue of DNDI-VL-2098 (14), a promising oral drug candidate for VL (15, 16) that was discontinued from further development due to toxicity issues identified during nonclinical clinical trial application-enabling studies (6). Given its superior safety profile, potent *in vitro* activity (50% effective concentration [EC_50_] = 0.17 μM), and excellent *in vivo* efficacy (>99% at 12.5 mg/kg of body weight orally twice a day in hamster models of VL) (17, 18), the decision was made in 2018 to progress DNDI-0690 into phase I clinical trials for VL. Furthermore, DNDI-0690 demonstrated excellent *in vitro* activity against three Old World and three New World cutaneous *Leishmania* strains (EC_50_ < 5 μM). In a mouse model of L. major CL, oral DNDI-0690 exerted a linear dose-response effect (50% effective dose = 5 mg/kg, 90% effective dose = 21 mg/kg, maximal efficacy > 95% for a dose of 50 mg/kg), while topical solutions applied directly to the skin lesion were <50% active (19).

With the clinical evaluation of DNDI-0690 for VL under way, important questions about the suitability, including appropriate pharmacokinetics (PK) and pharmacodynamics (PD), of this nitroimidazole compound in the treatment of CL remain. The PK and PD properties required of a drug to cure the two forms of leishmaniasis are not the same, due to (i) the different sites of infection that are the target for drug delivery (liver, spleen, and bone marrow versus dermal skin layers), (ii) possible differences in drug susceptibility between the causative parasites (L. donovani and L. infantum versus L. major, L. mexicana, and other dermatropic *Leishmania* species) (20), and (iii) the potential impact of pathology on drug distribution. The aim of this study was to evaluate the PK and PD parameters of DNDI-0690 as part of efforts to develop much-needed new oral or topical drugs to treat CL. We therefore determined the following properties of DNDI-0690: (i) *in vitro* drug disposition in skin upon topical dosing (Franz diffusion cells), (ii) *in vitro* protein binding (bicinchoninic acid [BCA] protein assay) and protein-binding-corrected 50% active drug concentrations against different CL-causing *Leishmania* species (free drug EC_50_ [*f*EC_50_]), (iii) *in vivo* protein-free (e.g., pharmacologically active) drug exposure at the dermal site of infection (microdialysis), and (iv) *in vivo* time-kill kinetics of L. major and L. mexicana (bioluminescent parasite imaging).

## RESULTS

### *In vitro* topical drug penetration.

First, we evaluated topical drug penetration of DNDI-0690 into mouse skin *in vitro* using Franz diffusion cell permeation assays to investigate why the topical application of DNDI-0690 led to limited antileishmanial activity in murine models of CL (19). Table 1 shows the skin distribution of topical DNDI-0690 into healthy and diseased but visibly intact skin (average nodule diameter, 4.10 ± 0.72 mm) harvested from L. major-infected BALB/c mice. Six hours after application of a solution of DNDI-0690 saturated in propylene glycol-ethanol (PG-EtOH; 0.063% [wt/vol]), about 99.5% of the drug remained on the skin surface. Only a limited amount of drug (0.07% to 0.34%) penetrated into the deeper layers of the (epi)dermis and 0.15% to 0.03% passed through the skin, indicating poor dermal retention. There was no significant difference in the drug quantity found in the different layers of the skin between L. major-infected and uninfected skin (*P* > 0.05).

**TABLE 1:** Disposition of topically applied DNDI-0690 in the skin of L. major-infected BALB/c mice using Franz diffusion cells

DNDI-0690 localization	Avg % recovered (±SD)^*a*^		*P* value^*b*^
	Uninfected skin	Infected skin	
On skin (DNDI-0690 in wash and cotton swab)	99.63 (±0.39)	99.77 (±0.19)	0.49
In skin (DNDI-0690 extracted from skin homogenate)	0.34 (±0.39)	0.07 (±0.07)	0.16
Through skin (DNDI-0690 in receptor fluid)	0.03 (±0.05)	0.15 (±0.15)	0.227

a The total amount of DNDI-0690 per Franz diffusion cell recovered at the end of the experiment was considered 100%. The amounts of DNDI-0690 recovered from the different sites were expressed as a fraction of this amount. The average (±SD) percent for 5 infected mice and 4 uninfected mice is shown.

b *P* values were determined by a *t* test.

### *In vitro* antileishmanial drug activity corrected for protein binding.

Second, the *in vitro* 50% effective concentrations (EC_50_) against *Leishmania* corrected for protein binding (*f*EC_50_) were calculated. This was done to enable comparison between *in vitro* PD measures (EC_50_ value based on the total drug concentrations in the drug assay medium) and *in vivo* PK parameters obtained by microdialysis (non-protein-bound drug concentrations). Therefore, protein binding in the *in vitro* assay medium (RPMI 1640 medium containing 10% heat-inactivated fetal calf serum [HiFCS]) was estimated using a rapid equilibrium method. Drug-protein binding in the medium was moderate: 45.8% at 0.2 μM DNDI-0690 and 53.1% at 1 μM DNDI-0690. The mean percent binding for DNDI-0690 (49.6%) was used to determine the *f*EC_50_, on the basis of previously obtained EC_50_ values (19) and as described in Table 2.

**TABLE 2:** *f*EC_50_ of DNDI-0690 against several cutaneous *Leishmania* species

CL-causing *Leishmania* species	*n*^*a*^	EC_50_ (μM)	*f*EC_50_ (μM)
L. major (MHOM/SA/85/JISH118)	1	4.56	2.28
	2	7.94	3.97
L. tropica (MHOM/AF/2015/HTD7)	1	1.41	0.71
	2	2.38	1.19
L. aethiopica (MHOM/ET/84/KH)	1	24.61	12.31
	2	<0.33	<0.165
L. mexicana (MNYC/BZ/62/M379)	1	1.91	0.96
	2	<1.11	<0.56
L. panamensis (MHOM/PA/67/BOYNTON)	1	0.77	0.39
L. amazonensis DsRed2	1	<1.11	<0.56

a *n*, number of experiment repeats.

### *In vivo* skin pharmacokinetics.

**FIG 2:**
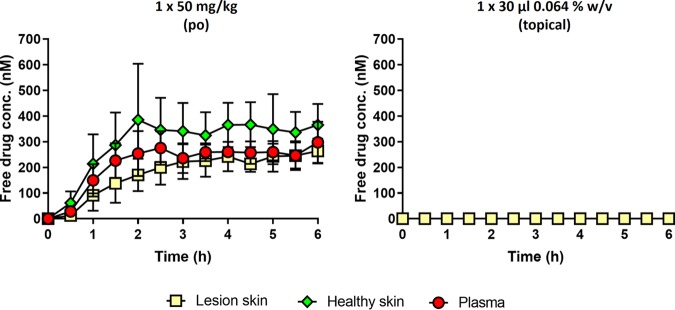
Skin PK of DNDI-0690 in the L. major-BALB/c mouse model of CL after oral drug administration (left; each mouse had 3 probes inserted, in the tail vein and healthy and lesion skin) and topical drug administration (right; each mouse had 1 probe inserted in the lesion skin). Data represent protein-free drug concentrations (average concentration ± SD [*n* = 3]), corrected for probe recovery.

Third, we studied the *in vivo* skin PK of DNDI-0690 in the L. major-BALB/c mouse model using microdialysis. After administration of either a single oral (50-mg/kg) or topical (30-μl, 0.063% [wt/vol]) dose of DNDI-0690 to the infected mice, free drug concentrations in the infected dermis (target site), the uninfected dermis (off-target site), and plasma (systemic circulation) were determined (Fig. 2). After oral dosing at 50 mg/kg, DNDI-0690 showed a gastrointestinal absorption delay of 2.5 h before reaching a free drug maximum concentration in plasma (*fC*_max_) of 275.4 ± 37.9 nM in the blood. Systemic drug concentrations remained stable in the following 3.5 h, indicating a plasma half-life (*t*_1/2_) of >4 h. The distribution volume (*V*) and elimination rate constant (*k*_el_) values could not be estimated because no significant clearance of DNDI-0690 from plasma occurred within 6 h of oral drug administration (the time that the last concentration was measured [*t*_last_]). The concentration of unbound DNDI-0690 in plasma was similar to the unbound drug concentrations in infected and uninfected skin tissue and followed a comparable trend. Drug penetration from blood into skin tissue was high (ratio of the area under the curve [0 to 6 h] of the free DNDI-0690 concentration in skin tissue to blood [*f*AUC_0-6 h, skin tissue_/*f*AUC_0-6 h, blood_] is greater than 80%) and maximal after 6 h of oral dosing. However, DNDI-0690 concentrations and the overall drug distribution to cutaneous tissues were increased in uninfected skin in comparison to that in infected skin (maximum concentration in plasma [*C*_max_] = 365.3 ± 47.1 nM versus 263.7 ± 28.0 nM, respectively; AUC_0–6 h, tissue_/AUC_0–6 h, blood_ = 136.7% versus 82.1%, respectively). In contrast, after topical application of 50 μl DNDI-0690 saturated solution to the lesion, no drug was detected in the infected dermis within the following 6 h. All results shown are corrected for the *in vitro* relative recovery (RR) of DNDI-0690 from the microdialysis probe. The RR was 18.6% ± 2.3% and independent of the concentration under *in vitro* experimental conditions mimicking those *in vivo*.

### *In vivo* antileishmanial pharmacodynamics.

**FIG 3:**
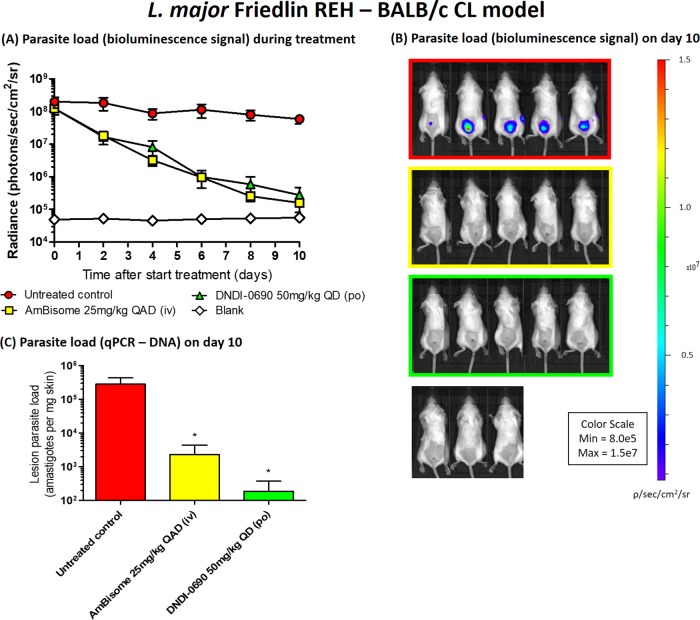
Antileishmanial efficacy of oral DNDI-0690 (50 mg/kg once daily for 10 days) in an Old World CL model (L. major Friedlin REH infection of BALB/c mice). (A) The parasite load, as indicated by *in vivo* imaging of bioluminescent parasites in the infected rump skin over time. (B) The bioluminescence signal on day 10 (24 h after the last drug dose administration). (C) The parasite load on day 10 was confirmed using qPCR. QD, once daily; QAD, once every 2 days; po, oral drug administration; iv, intravenous drug administration. *, *P* > 0.05.

**FIG 4:**
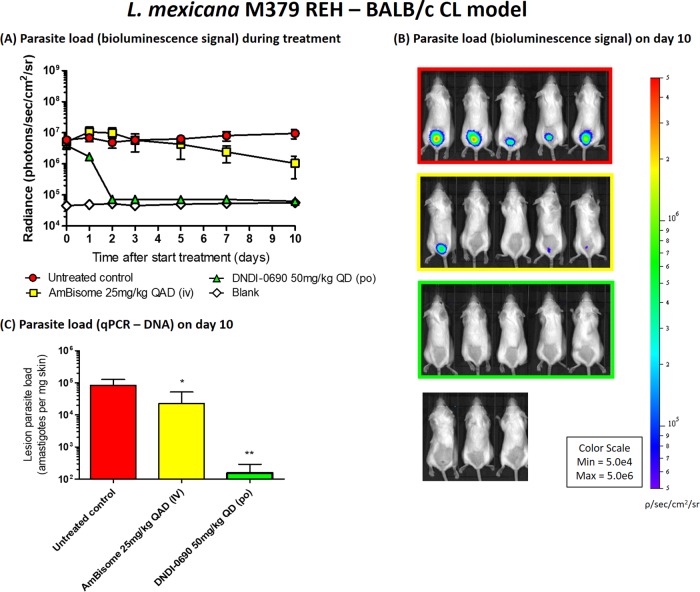
Antileishmanial efficacy of oral DNDI-0690 (50 mg/kg once daily for 10 days) in a New World CL model (L. mexicana M379 REH infection of BALB/c mice). (A) The parasite load, as indicated by *in vivo* imaging of bioluminescent parasites in the infected rump skin over time. (B) The bioluminescence signal on day 10 (24 h after the last drug dose administration). (C) The parasite load on day 10 was confirmed using qPCR. QD, once daily; QAD, once every 2 days; po, oral drug administration; iv, intravenous drug administration. *, *P* < 0.05; **, *P* < 0.01.

Fourth, the time-kill kinetics of DNDI-0690 were characterized in two BALB/c mouse models of CL using bioluminescent L. major and L. mexicana parasites. The topical activity of DNDI-0690 was not evaluated due to poor skin permeation and low efficacy when administered via this route. After oral dosing of DNDI-0690 (50 mg/kg once daily for 10 days), the rapid and complete clearance of L. major and L. mexicana from the infected mice was observed (Fig. 3 and 4, respectively). A 10-, 100-, and close to 1,000-fold reduction in the L. major parasite load (relative to the organism burden in untreated mice at the same time point) was observed by days 2, 6, and 10, respectively. The maximal killing of L. major (99.5%) was achieved 24 h after the 10th and final dose of DNDI-0690 (day 10). At this point, the efficacy of DNDI-0690 was comparable to that of the positive-control drug intravenous liposomal amphotericin B (LAmB; 99.7%) in this model. An identical regimen of once daily 50 mg/kg DNDI-0690 resulted in a 100-fold reduction in the L. mexicana parasite burden by day 2. After two oral doses, the bioluminescent signal in the DNDI-0690-treated group was indistinguishable from that in the mice infected with wild-type, nonbioluminescent parasites. The activity of DNDI-0690 against L. mexicana was maximal (99.4%) and higher than that of LAmB (89.0%) at the end of treatment (day 10). Quantitative PCR (qPCR) was used to confirm the >99% reductions in parasite load for L. major- and L. mexicana-infected mice treated with oral DNDI-0690 compared to that in the untreated controls (Fig. 3C and 4C, respectively).

## DISCUSSION

We have demonstrated the potential of DNDI-0690 as a novel treatment for CL by the oral route and the limited potential for its topical application. After oral administration at 50 mg/kg, DNDI-0690 is rapidly absorbed into the bloodstream and completely distributed to the skin, reaching nearly maximal drug exposure at the site of action within 3 h. At the dermal infection site, *fC*_max_ was lower (0.26 ± 0.03 μM) than the *f*EC_50_ values for all tested *Leishmania* species (0.4 to 12 μM), indicating that multiple doses might be needed to allow drug distribution to infected tissues and achieve cure. *In vivo* time-kill studies confirmed that this was the case; in order to obtain a 100-fold reduction in lesion parasite load, 2 doses of 50 mg/kg were needed to clear L. mexicana (*f*EC_50_ = 0.96 μM), whereas 6 doses were needed to clear L. major (*f*EC_50_ = 3.15 μM). In both these bioluminescent *Leishmania* parasite CL mouse models, oral DNDI-0690 was as efficacious as the intravenous antileishmanial drug LAmB at the end of the 10-day treatment (>99%). In contrast, topical administration of DNDI-0690 as a single application to the skin lesion did not result in measurable drug levels in the infected dermis. This may explain the low efficacy (<50% reduction of lesion size and parasite burden, as determined by qPCR) of treatment via this route seen in earlier studies (19). These poor *in vivo* drug penetration kinetics, determined by skin microdialysis, were successfully predicted by *in vitro* Franz diffusion cell assays, which revealed the inability of DNDI-0690 to permeate the epidermis (>99% drug was recovered from the skin surface). Such assays therefore save time and resources for the design and development of new topical formulations to treat simple CL (21).

To the best of our knowledge, this is the first time that skin microdialysis has been used to evaluate PK in *Leishmania*-infected mouse skin. The main technical advantage of this method for *in vivo* CL drug research is that it continuously measures protein-free (and, thus, pharmacologically active) drug concentrations directly in the dermal interstitial fluid surrounding the parasitized macrophages (22). Voelkner and colleagues employed a similar approach to evaluate the proposed antileishmanial drug pyrazinamide, although this experiment was performed in healthy rats (23).

Interestingly, we observed differences in the PK of oral DNDI-0690 in *Leishmania*-infected and uninfected skin. Inflammation at the infection site in CL affects the local drug distribution after intravenous administration of different formulations of amphotericin B (24, 25), as well as of topical drugs (26). Unbound DNDI-0690 concentrations in the dermal interstitial fluid could be lower in diseased skin than in healthy skin, because while larger absolute amounts of drug may reach the skin tissue from the bloodstream (increased vascular permeability, vasodilation) (25), more drug may be bound to inflammatory proteins or engulfed by macrophages in the dermis. As neither protein-bound nor intracellular drug fractions are measured by microdialysis (27), this could explain the ultimately lower extracellular exposure of DNDI-0690 at the site of infection compared to that in the uninfected counterparts. This finding illustrates the impact of the CL pathology on the local drug distribution in the skin. Differences between amphotericin B and DNDI-0690 PK results could be related to the different sampling methodologies (skin necropsies and microdialysis, respectively).

A limitation of this work is the single, high dose of oral DNDI-0690 (50 mg/kg) that was used during the PK and PD experiments. Further dose fractionation studies are required to identify the PK/PD driver of efficacy in CL (28). Combined with extended PK studies in mice and humans (different dose levels and time points of >6 h), available data on the susceptibility of six parasite species and strains to DNDI-0690 can be used to set a robust PK/PD target estimate to inform the design of optimal clinical dosing regimens.

In conclusion, the rapid oral absorption and potent activity of DNDI-0690 in skin lesions caused by L. major and L. mexicana support further development of this preclinical drug candidate as a new oral treatment for CL.

## MATERIALS AND METHODS

### Drugs and reagents.

Oral DNDI-0690 was formulated in polyethylene glycol 400 (PEG400). The suspension was prepared in glass vials containing glass beads and sonicated (CamLab, Cambridge, UK) for 15 min before use. The dose levels and dosing frequency chosen were based on efficacy observed against VL (18) and CL (20). Topical DNDI-0690 was formulated as a saturated solution in propylene glycol-ethanol (PG-EtOH; 1:1) to maximize permeation through the skin. The preparation was as follows. Excess drug compound was added to a glass vial together with 1 ml of PG-EtOH (1:1) and a magnetic stirrer. The vial was covered with aluminum foil and left at 34°C for 24 h. An aliquot of this suspension was transferred to a vial and centrifuged for 15 min at 18,407 × *g* and 34°C, after which the supernatant was transferred to a clean vial and stored at 4°C until drug administration. Liquid chromatography-tandem mass spectrometry (LC-MS/MS) analysis confirmed the concentration of DNDI-0690 in this topical vehicle to be 0.063% (wt/vol). Ringer’s solution was prepared at full strength (Sigma-Aldrich), dissolved in 500 ml purified water, and autoclaved before use.

### Parasite maintenance, animals, and ethical statement.

The bioluminescent strains *Ppy RE9H+*L. major Friedlin (MHOM/IL/81/Friedlin) and *Ppy RE9H+*L. mexicana M379 (MNYC/BZ/62/M379) were kindly provided by Elmarie Myburgh and Jeremy Mottram (University of York, York, UK). The L. major JISH wild-type (WT) strain (MHOM/SA/85/JISH118), *Ppy RE9H+*L. major Friedlin, and *Ppy RE9H+*L. mexicana M379 were maintained in Schneider’s medium supplemented with 10% heat-inactivated fetal calf serum (HiFCS) and passaged weekly (1:10). Female BALB/c mice (age, 6 to 8 weeks) were purchased from Charles River (Margate, UK) and left to acclimatize for 5 days upon arrival. One day prior to infection, the rump above the tail was shaven using electric clippers. Twenty-four hours later, low-passage late-stationary-phase promastigote cultures were centrifuged at 900 × *g* for 10 min at 4°C, and the promastigotes were counted using an improved Neubauer hemocytometer and resuspended to 2 × 10^8^ promastigotes per ml. Mice were subcutaneously injected in the rump with 200 μl of the suspension and randomly grouped (*n* = 3 to 5). The mice were housed in a controlled environment of 55% relative humidity and 26°C and provided with tap water and a standard laboratory diet. All *in vivo* experiments were carried out under license (X20014A54) at the London School of Hygiene and Tropical Medicine (LSHTM) after discussion with the named veterinarian surgeon and according to UK Home Office regulations.

### Bioanalysis of DNDI-0690 (LC-MS/MS).

All samples were analyzed, using a Shimadzu Nexera X2 ultra-high-performance liquid chromatograph and a Shimadzu LCMS 860 mass spectrometer, at Pharmidex Pharmaceutical Services Ltd. (Hatfield, UK). A mobile phase (0.4 ml/min) of water–0.1% formic (channel A) and acetonitrile–0.1% formic acid (channel B) was used to elute the sample compound from a Kinetex 5-μm XB-C_18_ column (2.1 mm by 50 mm at 50°C; Phenomenex, UK). The mobile phase composition was initially 2% channel B programmed to increase linearly to 95% channel B at 1.1 min after injection. After 0.7 min at 95% channel B, the composition was returned to its initial 2% channel B at 1.8 min postinjection. DNDI-0690 was detected by monitoring the transition of the parent molecule (mass-to-charge ratio [*m/z*] 370) to the fragment resulting from electrospray ionization (*m/z* 198.2). Analyte concentrations were quantified against calibration standards prepared in matched control matrices, with aliquots of samples, blanks, and standards being injected at 5 μl. The lower limit of quantifications ranged from 0.5 ng/ml to 50 ng/ml for the microdialysis and skin extraction samples, respectively (see the additional information on the LC-MS method in the supplemental material).

### *In vitro* drug binding.

The *in vitro* binding of the drug compounds to skin components was measured using a rapid equilibrium dialysis (RED) single-use device (Pierce Red device; Thermo Scientific). A 20 mM solution of DNDI-0690 in dimethyl sulfoxide was used to spike RPMI 1640 medium supplemented with 10% HiFCS to a final concentration of 0.2 and 1 μM DNDI-0690. Three hundred microliters of the DNDI-0690-containing medium was transferred to the sample chamber, and 550 μl of Ringer’s solution was added to the buffer chamber. This was done in triplicate for each DNDI-0690 concentration. The RED device was left to incubate in an orbital shaking incubator (200 rpm) at 34°C for 4 h. From each chamber, 50-μl aliquots were collected and matrix matched, after which 2 volumes of ice-cold acetonitrile [ACN] was added. After another 20 min, 100 μl of each mixture was centrifuged for 15 min at 21,130 × *g* at 4°C. The supernatants were assayed for the parent drug by LC-MS/MS.

### Franz diffusion cell permeation and drug disposition.

Female BALB/c mice (*n* = 5) were injected subcutaneously with 4 × 10^7^ L. major promastigotes above the tail. In time, a nodule developed at the injection site, and when this reached 4 to 5 mm, the mice were sacrificed using CO_2_. Two circular skin discs (approximately 15 mm in diameter) were obtained per donor mouse; one contained the leishmaniasis nodule (average ± standard deviation [SD]) that was collected from the lower dorsal area above the tail, and another disc of unaffected skin was collected from the area higher up the back of the mouse. Fat and muscle tissue were carefully removed using forceps, and the skin was gently stretched on Whatman filter paper. The skin was placed between the greased donor and receptor compartment of the Franz cell with a narrow diameter (5 mm). Phosphate-buffered saline (PBS) was sonicated for 30 min and added to the receptor compartment together with a small magnetic stirrer. The Franz cells were placed on the magnetic stirrer plate (800 rpm) in a warm water bath until the skin temperature reached a steady 34°C. Next, the DNDI-0690 saturated solution (30 μl of 0.063% [wt/vol] DNDI-0690 in PG-EtOH [1:1]) was applied to the skin, and 100 μl of receptor solution was replaced with fresh PBS at regular time intervals over a period of 6 h and analyzed by LC-MS/MS. At the end of the experiment, the cells were dismantled and the donor chambers of the Franz cells were washed with 1 ml of acetonitrile-water solution (ACN-H_2_O [1:1]). Any drug remaining on the skin surface was removed using a clean dry cotton swab. The amount of drug in the washing liquid and the cotton swab was quantified using LC-MS/MS. To extract DNDI-0690 from the skin, the skin disc was homogenized in 1 ml of PBS as described above. One hundred microliters of this homogenate was protein precipitated using 300 μl of ice-cold ACN (100%) and centrifuged at 13,000 rpm for 30 min at 4°C. An aliquot of the supernatant was diluted with an equal volume of water and stored for analysis by LC-MS/MS at −80°C. Together, the amount of drug that was recovered from the skin surface, that was extracted from the skin, and that permeated through the skin was satisfactory when it ranged from 70% to 110%.

### Microdialysis system.

MAB 1.2.4.Cu probes (Microbiotech, Sweden) with a 6-kDa-cutoff cuprophane membrane were used *in vitro* for recovery determination and *in vivo* for microdialysis. The cuprophane membrane of this concentric probe is 2 mm long and has an outer membrane diameter of 0.2 mm; inlet and outlet tubing consisted of fluorinated ethylene propylene (FEP) with lengths of 25 and 50 cm, respectively. A syringe pump (11 plus model 70-2208; Harvard Apparatus, USA) was used to circulate the perfusate (Ringer’s solution) at a flow rate of 2 μl/min. Dialysates were automatically collected in glass vials (Thermo Fisher, UK) using a refrigerated fraction collector (MAB 85; Microbiotech, Sweden) at 30-min set intervals. For accurate measurement of *in vivo* free drug concentrations at the dermal site of action, raw microdialysis values were corrected for the loss of compound due to incomplete equilibration between the sampling medium and the perfusate and/or sticking of the drug to the outlet tubing of the microdialysis probe, expressed as the relative recovery value (22). Recovery rates for the microdialysis equipment were determined *in vitro* as follows: three probes were placed in a reservoir containing DNDI-0690 at a concentration of 30 or 120 ng/ml in Ringer’s solution at 34°C (mimicking the *in vivo* skin temperature). The probes were perfused with Ringer’s solution at a flow rate of 2 μl/min, and microdialysates were collected every 15 min. All samples were analyzed using LC-MS/MS after the addition of 10 μl acetonitrile (ACN; 1:3 ratio for a 30-μl sample volume). Relative recovery (RR) was calculated as the ratio of the analyte concentrations in the microdialysate over the analyte concentration in the reservoir medium.

### *In vivo* microdialysis.

**FIG 5:**
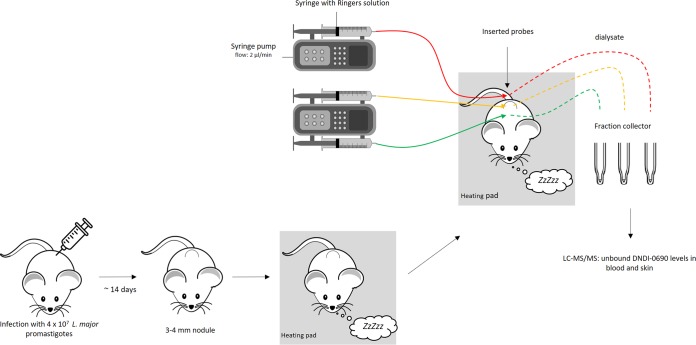
Schematic of the experimental setup of *in vivo* microdialysis in mice with CL.

L. major JISH-infected BALB/c mice (*n* = 6) with a shaven rump and back were anesthetized with 1.6 g/kg urethane (intraperitoneally). Two hundred microliters of Ringer’s physiological solution was immediately administered via the neck scruff (subcutaneously) to prevent dehydration during long-term (6- to 8-h) anesthesia. Mice were placed on a temperature-controlled heating pad (Peco Services Ltd., Cumbria, UK) to maintain the body temperature at 32 ± 2°C. MAB 1.2.4 Cu probes were inserted in the following positions using a 22-gauge needle: the dermal skin layer of the CL lesion on the rump, the dermal skin layer of the healthy control skin higher up on the back, and the tail vein (Fig. 5). To equilibrate the system and allow the skin and tail vein to recover from the probe insertion trauma, a stabilization period of 30 min (23) of perfusion with Ringer’s solution at a flow rate of 2 μl/min was included before samples were collected. At the start of the pharmacokinetic experiment, half of the mice (*n* = 3) received 50 mg/kg DNDI-0690 via oral gavage. This dosage has been shown to significantly reduce the lesion size (20). The other three mice received 30 μl of a 0.063% (wt/vol) saturated solution (maximal driving force, 1) applied topically to the skin lesion on the rump of the mice. Samples were collected every 30 min at a flow rate of 2 μl/min. After the addition of 20 μl acetonitrile (1:3 ratio for a 60-μl sample volume), samples were stored at –80°C before analysis by LC-MS/MS. The temperature, breathing pattern, and behavior of the anesthetized mice were monitored constantly. At the end of the experiment, the mice were culled by pentobarbital overdose.

Single-dose PK parameters were calculated by plotting the DNDI-0690 concentrations measured in the dialysate of the probe placed in the blood vene and the infected and uninfected skin over time. The *fC*_max_ for each matrix (blood, infected and uninfected skin) was the highest drug concentration reached in each respective matrix throughout the experiment. *f*AUC_0–6 h_ values for the blood and infected and uninfected skin were calculated using GraphPad Prism (version 7.02) software. Data are presented as the mean and standard error of the mean (SEM).

### Rate of kill by *in vivo* bioluminescence imaging.

Thirty-six female BALB/c mice were purchased and prepared for infection as described above. Fifteen mice were injected with 4 × 10^7^ stationary-phase luciferase-expressing L. major Friedlin (*Ppy RE9H+*L. major Friedlin) promastigotes, 15 were injected with luciferase-expressing L. mexicana M379 (*Ppy RE9H+*L. mexicana M379) parasites (23), and 6 were infected with L. major JISH WT parasites, which do not express luciferase. Upon appearance, nodule diameters were measured in two directions daily. When the size progressed to 6.73 ± 1 mm for the L. major Friedlin-infected mice, they were allocated into groups of five with similar average nodule diameters (*P* > 0.5, one-way analysis of variance [ANOVA]), and treatment was initiated. For the mice infected with *Ppy RE9H+*L. mexicana M379, no lesions developed and treatment was started when the bioluminescence signal reached 5.02 × 10^6^ ± 3.27 × 10^6^ radiance/second (where radiance is the number of photons per second per square centimeter per steradian [sr]). Each experiment included an untreated control group (*n* = 5), a baseline control group (L. major JISH WT, *n* = 3), a positive-control group (liposomal amphotericin B for injection [AmBisome], intravenously, 25 mg/kg, once every 2 days, *n* = 5), and a DNDI-0690-treated group (50 mg/kg, orally, once a day, *n* = 5). A topical administration group was not included due to the previously observed inactivity. The bioluminescent signal was measured prior to administration of the first drug dose and every other day thereafter until the baseline signal was reached. Ten minutes before acquiring the bioluminescent signal, mice were injected with 150 mg/kg luciferin (d-luciferin potassium salt; Bertin Bioreagent) and then anesthetized using 3% (vol/vol) gaseous isoflurane and placed in an IVIS Lumina II system (PerkinElmer). Images were acquired 10 min after luciferin injection using Living Image (version 4.3) software. A circular region of interest (ROI) encompassing the nodular area on the rump was drawn to quantify the bioluminescence, expressed as radiance. Background radiance was measured from mice infected with the L. major JISH WT. The parasite burden in the skin was confirmed by DNA-based qPCR, as described earlier (24).

### Statistical analysis.

For the *in vitro* topical drug penetration experiment, differences between the DNDI-0690 concentrations in healthy and infected skin were compared using Student's *t* test (Prism [version 7.02] software; GraphPad). To compare differences in the parasite load in skin lesions determined by qPCR, one-way analysis of variance (ANOVA) followed by Dunnett’s multiple-comparison test was performed. A *P* value of <0.05 was considered statistically significant.

## Supplementary Material

Supplemental file 1
