# Supplementary material for: Pharmacokinetics and Pharmacodynamics of the Nitroimidazole DNDI-0690 in Mouse Models of Cutaneous Leishmaniasis
Source: Antimicrob Agents Chemother. 2019 Aug 23;63(9):e00829-19. doi: 10.1128/AAC.00829-19 (PMC6709472; doi:10.1128/AAC.00829-19)
Supplement: Supplemental file 1 [file AAC.00829-19-s0001.pdf]

Supplemental Material 1 – Additional Information LC-MS method

Instrument and method information

Instrument Shimadzu Nexera X2 UHPLC/Shimadzu LCMS 860

MS conditions ESI: Positive ion electrospray

parent molecule m/z	370.0
fragment molecule m/z	198.2

UPLC conditions Mobile Phase:

Mobile phase A: Water, 0.1% formic acid

Mobile phase B: Acetonitrile, 0.1% formic acid

Time (min)	Mobile Phase B (%)
0	2
0.3	2
1.1	95
1.75	95
1.8	2
2	2

Column: Kinetex 5-µm XB-C18 column (21x50mm)
Column temperature: 50°C
Flow rate: 0.4 mL/min
Injection volume: 5ul
Divert to waste: 0-0.6 min

Evaluation

Analyte	Matrix	LLOQ	ULOQ	R^2 *	Accuracy
DNDI-0690 (m/z 371.10)	**Microdialysis work**				
	Dialysate	0.5	500	0.993	80-120%
	**Franz diffusion cell studies**				
	Wash fraction	20	2000	0.991	80-120%
	Permeation samples	20	2000	0.993	80-120%
	BALB/c skin	50	5000	0.960	80-120%

* A quadratic regression, with an equation $y = ax^2 + bx + c$, was used to fit calibration curves over the concentration range.

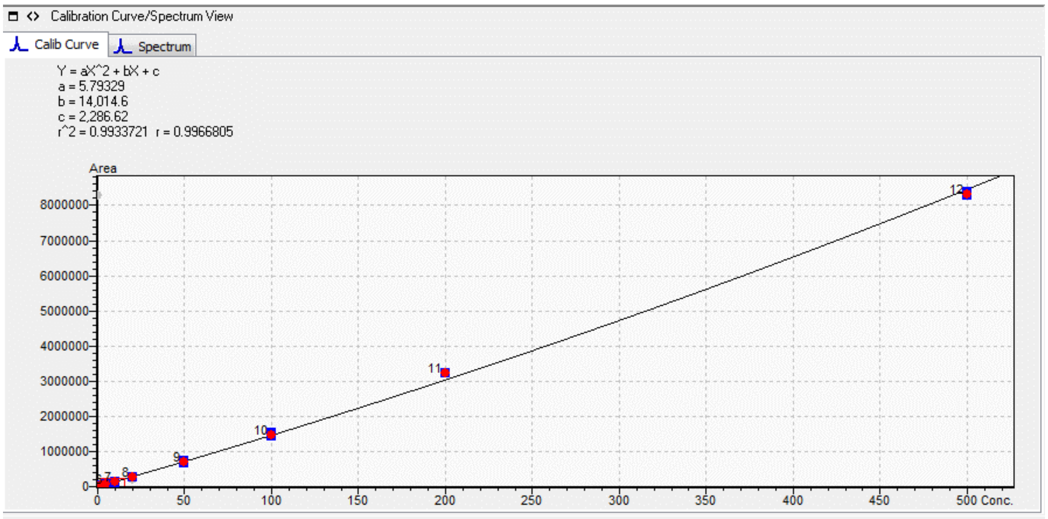

Figure S1. One of the calibration curves used to measure the DNDI-0690 concentration in skin microdialysate. Blank skin microdialysate was spiked with different concentrations of DNDI-0690 to obtain each standard.
